# Nurses’ Workplace Conditions Impacting Their Mental Health during COVID-19: A Cross-Sectional Survey Study

**DOI:** 10.3390/healthcare9010084

**Published:** 2021-01-16

**Authors:** Farinaz Havaei, Andy Ma, Sabina Staempfli, Maura MacPhee

**Affiliations:** School of Nursing, The University of British Columbia, Vancouver, BC V6T 1Z4, Canada; andytfma@mail.ubc.ca (A.M.); sabina.staempfli@ubc.ca (S.S.); Maura.MacPhee@ubc.ca (M.M.)

**Keywords:** nurses, mental health, work environment, COVID-19

## Abstract

Among health workers, nurses are at the greatest risk of COVID-19 exposure and mortality due to their workplace conditions, including shortages of personal protective equipment (PPE), insufficient staffing, and inadequate safety precautions. The purpose of this study was to examine the impact of COVID-19 workplace conditions on nurses’ mental health outcomes. A cross-sectional correlational design was used. An electronic survey was emailed to nurses in one Canadian province between June and July of 2020. A total of 3676 responses were included in this study. We found concerning prevalence rates for post-traumatic stress disorder (47%), anxiety (38%), depression (41%), and high emotional exhaustion (60%). Negative ratings of workplace relations, organizational support, organizational preparedness, workplace safety, and access to supplies and resources were associated with higher scores on all of the adverse mental health outcomes included in this study. Better workplace policies and practices are urgently required to prevent and mitigate nurses’ suboptimal work conditions, given their concerning mental health self-reports during the COVID-19 pandemic.

## 1. Introduction

Among health workers, nurses are at highest risk of COVID-19 exposure and mortality due to work environment conditions, including personal protective equipment (PPE) shortages, insufficient staffing, and inadequate safety training and preparation [1,2]. Early research suggests that nurses are a high-risk population for adverse mental health outcomes during the COVID-19 pandemic [3,4]. To our knowledge, this is the first Canadian study to examine the impact of COVID-19 workplace conditions on nurses’ mental health outcomes. The purpose of this study was to explore the association between nurses’ workplace conditions during COVID-19 and their mental health in a sample from one Canadian province. Evidence from this study will inform mental health promotion policies and interventions during a time when nurses are a rapidly dwindling health human resource [5,6].

### 1.1. Literature Review

Mental health is “a state of well-being in which an individual realizes his or her own abilities, can cope with the normal stresses of life, can work productively and is able to make a contribution to his or her community” [7]. In nursing research, mental health is often measured by the absence of mental health disorders, including post-traumatic stress disorder (PTSD), anxiety, depression, and burnout. PTSD can develop after a traumatic event and is characterized by hypervigilance and impaired concentration, including workplace avoidance behaviors and the presence of nightmares and flashbacks of the traumatic event [8]. Depression is associated with persistent feelings of sadness, hopelessness, and loss of interest [9]. Anxiety is described as persistent excessive and unnecessary worry about events or activities [10]. Burnout is characterized by high levels of emotional exhaustion (EE), depersonalization (DP), and reduced personal accomplishment (PA) [11]. In nursing research, EE is often used as the most important indicator of burnout [12,13].

The mental health status of the nursing workforce has been a chronic concern predating COVID-19. As early as 2005, a national survey of the work and health of Canadian nurses found one in six nurses reported that mental health problems interfered with their ability to work, and 10% of the sample met the criteria for depression [14]. Other studies found high prevalence rates of PTSD and burnout among Slovenian nurses [15]. A recent pre-COVID study in British Columbia, Canada, found that one-third of the sample of 5500 nurses met the criteria for anxiety and depression, and half the sample met the criteria for high EE and PTSD [16]. Given emerging global pandemic evidence of nurses’ worsening mental health and work environment concerns [2,3,4,17], we conducted a survey of nurses in one Canadian province to explore the association between their mental health and workplace conditions during the COVID-19 pandemic.

### 1.2. Theoretical Framework

Previous research demonstrates that workplace conditions are one of the most important predictors of nurse outcomes, including their mental health and wellbeing [18,19,20,21]. In particular, a 2018 meta-analysis of 17 studies using data from 2677 hospitals in 22 countries found that poor nursing work environments were associated with poor patient and nurse outcomes, including burnout [21]. Other researchers linked unhealthy nursing work environments to poor mental health, specifically anxiety, insomnia, and psychotropic medication use [12,22].

This study was informed by an adapted version of the nursing worklife model [20]. This model has been widely used to study relationships between five key domains in nurses’ work environments (i.e., staffing and resources, collegial workplace relations, leadership support, policy impact, and a nursing foundation of care) and patient and nurse outcomes [20,23,24]. For this study, we used three of the original domains (i.e., staffing and resource adequacy, workplace relations, and leadership/organizational support), and we adapted two domains for the COVID-19 context. Our adapted domains measured ensuring workplace safety and organizational preparedness. These adaptations reflect COVID-19 work environment safety issues and pandemic management planning [25,26].

## 2. Materials and Methods

### 2.1. Data Collection and Sample

All members (~48,000) of the provincial nurses’ union were invited to complete an electronic survey. An email invitation with the survey link was distributed by the union to its nurse members, and participation was encouraged through social media advertisement, email reminders, and a raffle draw for ten $100 prepaid Visa cards. Participants were fully informed of the confidentiality of their responses and the voluntary nature of survey participation. They were also informed that survey completion and submission would indicate informed consent. The data collection interval was from June 2020 to July 2020. A total of 4523 survey responses was received yielding a response rate of about 10%. For this study, we included only actively working nurses (*n* = 3676). Ethics approval was granted by the University Behavioral Research Ethics Board (H20-01861).

### 2.2. Measures

#### 2.2.1. Outcome Variables

Post-traumatic stress disorder was measured using a validated scale, the Posttraumatic Stress Symptoms-14 (PTSS-14) instrument [8]. The PTSS-14 is comprised of 14 items that reflect symptoms as described by the Diagnostic and Statistical Manual of Mental Disorders IV (DSM-IV) criteria for PTSD, such as feelings of guilt or nightmares about duty in the primary workplace. Respondents’ feelings over the last two weeks were rated on a 7-point Likert-type scale ranging from 1 (never) to 7 (always). Sum scores were tallied from each participant’s responses, with prior research establishing a cutoff score of 45 as an indicator of PTSD. An exploratory factor analysis (EFA) with varimax rotation demonstrated a unidimensional factor structure (forced) explaining 55% of the variance among the study sample (factor loadings: 0.60–0.80, α = 0.94).

Anxiety was assessed using the validated Generalized Anxiety Disorder-7 (GAD-7) instrument, which includes seven items describing symptoms of generalized anxiety disorder as outlined by the DSM-IV diagnostic criteria [10]. Respondents were asked to rate the frequency of the feelings described over the last two weeks along a 4-point Likert-type scale ranging from 0 (not at all) to 3 (nearly every day). Examples of items include trouble relaxing, not being able to stop or control worrying, and becoming easily annoyed or irritable. Spitzer and colleagues found a cutoff sum score of 10 or greater to identify anxiety with the greatest sensitivity (89%) and specificity (82%) [10]. In this study, the measure demonstrated a unidimensional factor structure explaining 71% of the variance using EFA with varimax rotation (factor loadings: 0.76–0.90, α = 0.93).

Depression was measured using the Patient Health Questionnaire-9 (PHQ-9) [9]. The PHQ-9 consists of nine items reflecting feelings, meeting the DSM-IV diagnostic criteria for depression, such as depressive mood, poor appetite, and insomnia. Respondents rated how often they experienced these symptoms over the past two weeks, along a 4-point Likert-type scale ranging from 0 (not at all) to 3 (nearly every day). Kroenke et al. found a cutoff sum score of 10 or greater to capture major depression with the greatest sensitivity (88%) and specificity (88%) [9]. An EFA with varimax rotation showed a unidimensional factor structure explaining 56% of the variance among the study sample (factor loadings: 0.53–0.83, α = 0.90).

Finally, EE was measured using items from the EE subscale of the Maslach Burnout Inventory–Human Services Survey (MBI-HSS). Items from the subscale include statements such as “I feel emotionally drained from my work” and “I feel like I’m at the end of my rope”, with respondents rating the frequency of the described feeling along a 7-point Likert-type scale ranging from 0 (never) to 6 (every day) [27]. The sum scores for the EE subscale were categorized by cutoff scores into the following categories: 0–16 = low, 17–26 = moderate, ≥27 = high EE [27]. An EFA with varimax rotation showed a unidimensional factor structure explaining 63% of the variance among the study sample (factor loadings: 0.66–0.88, α = 0.93).

#### 2.2.2. Key Predictors

**Figure 1:**
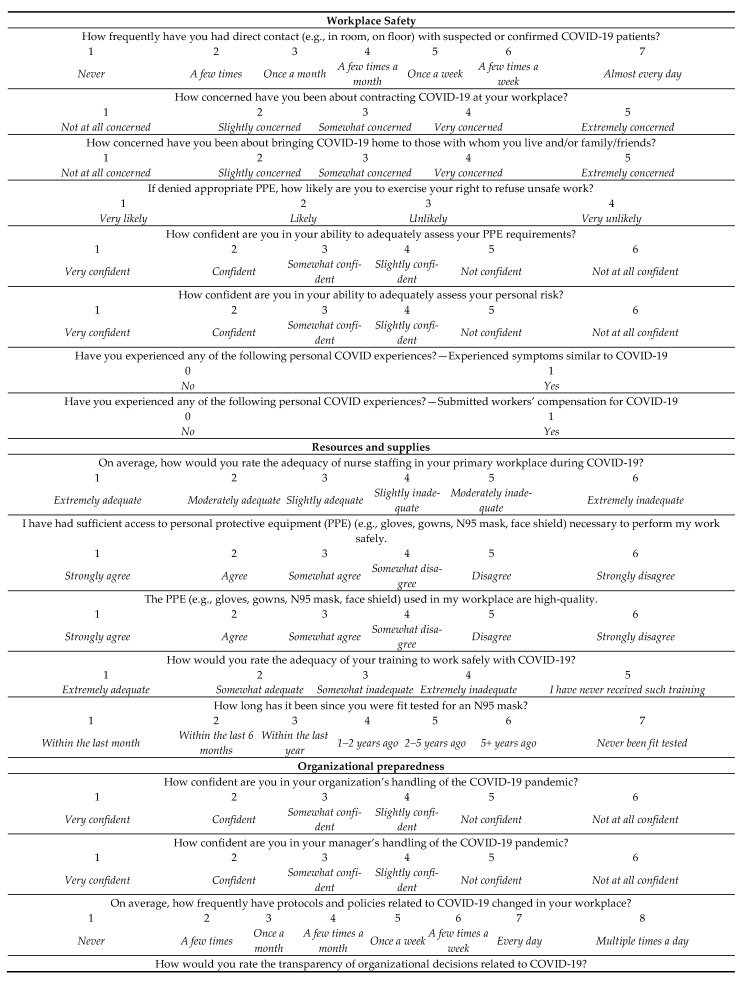

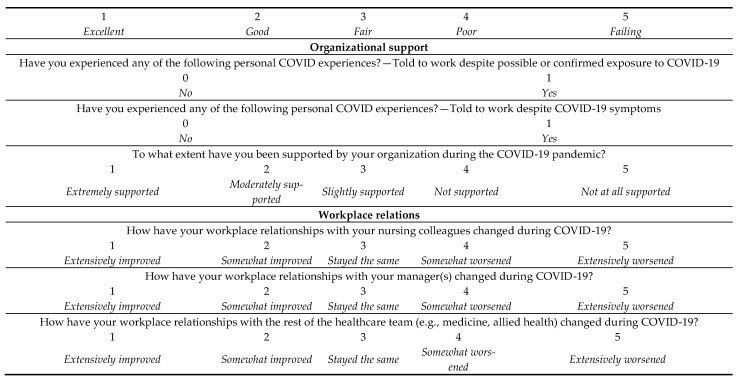
Workplace conditions questions and their response scales for each domain. PPE = personal protective equipment.

Nurses’ workplace conditions during COVID-19 were captured using a series of 23 questions spanning the five domains of *workplace safety* (8 items, e.g., concern about exposure to COVID-19 at work), *access to resources and supplies* (5 items, e.g., adequate access to PPE), *organizational preparedness* (4 items, e.g., confidence in organizational handling of the pandemic), *organizational support* (3 items, e.g., support from the organization), and *workplace relations* (3 items, e.g., change in relations with nursing colleagues) during the pandemic. The complete list of items per domain and their response scales can be found in Figure 1, along with their response options and coding. Questions for the two adapted domains, workplace safety and organizational preparedness, were from a recent survey conducted by the Institute of Work and Health [28]. Questions for other domains were adapted from the Nursing Work Index [20,21]. All questions were content validated with the provincial nurses’ union subject matter experts. Given the exploratory nature of this study, individual items from each domain were used as a predictor in our regression models and their Likert-type scales were treated as numerical predictors.

#### 2.2.3. Control Variables

Demographics were measured using a series of researcher-developed questions based on our previous research with nurses. We included questions on nurses (e.g., nursing experience, designation, role) and workplace characteristics (e.g., workplace geography, sector). Nurse designation included college-educated licensed practical nurse (LPN), baccalaureate-educated registered psychiatric nurse (RPN), and baccalaureate-educated registered nurse (RN). Response data were recoded from existing categories as binaries for all control variables except experience: workplace geography (0 = rural, 1 = urban/suburban), nursing designation (0 = LPN, 1 = RN/RPN/Dual), nursing role (0 = leader/educator, 1 = direct care provider), and sector (0 = community/long-term care, 1 = acute care).

### 2.3. Analysis

Key methods of data analysis included descriptive statistics and multiple linear regression using the Statistical Package for Social Sciences 27 (SPSS Inc., Chicago, IL, USA). To save power and create parsimonious models for all outcome variables, we started our regression analyses with four exploratory models including all workplace condition predictors. Predictors that were non-significant across all models were removed. Our final regression models included five control variables followed by 14 predictors in blocks of workplace safety (5 predictors), resources and supplies (2 predictors), organizational preparedness (3 predictors), organizational support (1 predictor), and workplace relations (3 predictors). Missing data were handled through listwise deletion in all models. Regression assumptions of linearity, independence, normality, equality of variance, and multicollinearity were examined through various model diagnostics and found to be satisfactory.

## 3. Results

A summary of response data for the demographic variables is shown in Table 1. The sample of actively working nurses in this study (*n* = 3676) consisted primarily of RNs/RPNs (80%) and LPNs (19%). The majority of the sample reported their nursing role as direct care provider (86%) and their geographic area as urban or suburban (84%). More than half indicated their nursing sector as acute care (63%). The mean for nursing experience was approximately 12 years (SD = 7.2), with 24% reporting 5 years of experience or less, while 28% reporting 21 years or more.

Table 2 shows descriptive statistics such as means and standard deviations for the COVID-19 workplace condition items. Overall, there were concerns related to most aspects of nurses’ workplace conditions during COVID-19. With respect to workplace safety, most importantly, 86% of the sample identified being somewhat to extremely concerned about brining COVID-19 home ($\bar{x}\;$= 3.90, SD = 1.14) and 80% about their own workplace exposure to COVID-19 ($\bar{x}$ = 3.47, SD = 1.12). With respect to resources and supplies, 52% reported inadequate nurse staffing ($\bar{x}$ = 3.48, SD = 1.60); 49% indicated some level of disagreement about access to high-quality PPE ($\bar{x}$ = 3.59, SD = 1.50) and 42% about access to sufficient PPE in their workplace ($\bar{x}$ = 3.30, SD = 1.54). With respect to organizational preparedness, 41% rated the transparency of organizational decisions related to COVID-19 as poor or failing ($\bar{x}$ = 3.29, *SD* = 1.02); and 27% reported daily or multiple times a day changes to COVID-19 related protocols and policies ($\bar{x}$ = 5.47, SD = 3.29). With respect to organizational support, 24% were told to work despite possible or confirmed exposure to the virus ($\bar{x}$ = 0.24, SD = 0.43); and 18% felt they did not receive any organizational support ($\bar{x}$ = 2.64, SD = 1.03) during COVID-19. Finally, with respect to workplace relations, 23%, 24%, and 31% reported worsening relations, respectively, with the rest of the allied health team ($\bar{x}$ = 3.11, SD = 0.70), nursing colleagues ($\bar{x}$ = 3.05, SD = 0.79), and management ($\bar{x}$ = 3.25, SD = 0.80). 

Table 3 shows response proportions and descriptive statistics for the measures used as mental health outcome indicators. Almost one-half of the sample (47%) met the diagnostic cutoff of 45 indicative of potential PTSD. Approximately two-fifths of the nurses (38%) met the criteria for anxiety and roughly the same proportion (41%) for major depression. Nearly two-thirds of nurses (60%) met the criteria for high EE.

### Regression Analysis Results

As regression analyses were conducted for each of the four outcomes, four final models were created, and the results are tabulated in Table 4. A number of predictors were removed during our modelling process due to their low predictive ability across all outcomes. Confidence in ability to assess PPE requirements, confidence in ability to assess risk, and experience of submitting workers’ compensation were removed from the workplace safety block. The variables for PPE quality, COVID-19 related training, and N95 mask fitting were removed from the resources and supplies block. Ratings of confidence in manager’s pandemic handling was removed from the organizational preparedness block, while the binary variables for experiencing “told to work despite exposure” and “told to work despite symptoms” were removed from the organizational support block.

All four final models regressing control variables and workplace condition predictors on the sum scores of four mental health measures were significant. Regression results are shown in Table 4. Adjusted *R*^2^ values indicate that the proportions of explained variance ranged from 19.0% (depression model) to 25.7% (PTSD and EE models). For all models, across all statistically significant predictors, more unfavorable perceptions of workplace conditions during COVID-19 were associated with greater scores on unfavorable mental health outcomes.

All variables within the workplace safety, resources and supplies, organizational support, and workplace relations blocks were statistically significant predictors of PTSD, while only the “frequency of COVID-19-related policy changes” from the organizational preparedness block was significantly related to PTSD scores. Affirmative responses on the dichotomous variable of “experiencing COVID-19 symptoms” predicted a 4.12 point increase (95% CI = 2.87–5.37, *p* < 0.001) in PTSD scores; other variables held constant. To illustrate the relation between Likert-type predictors and the outcome measure, a 1 point increase (indicating worsening) on the 6-point scale for “relationships with the rest of allied” was associated with a 2.44 point increase in PTSD scores (95% CI = 1.49–3.39, *p* < 0.001) after accounting for the other model variables.

With the exception of “frequency of direct contact” from the workplace safety block, “PPE access adequacy” from the resources and supplies block, “confidence in organizational handling of the pandemic” and “transparency of organizational decisions” from the organizational preparedness block, all other indicators of COVID-19 workplace conditions were significantly related to anxiety scores.

With the exception of “frequency of direct contact” and “concern about contracting COVID-19” from the workplace safety block, “confidence in organizational handling” and “transparency of pandemic related organizational decisions” from the organizational preparedness block, and “relationships with manager” from the workplace relations block, all other indicators of workplace conditions during COVID-19 were related to nurse depression scores.

Finally, while all indicators of organizational preparedness, organizational support, and workplace relations were positively related to EE scores, only “frequency of direct contact” and “experiencing COVID-19 symptoms” from the workplace safety block and “nurse staffing adequacy” from the resources and supplies block were significantly related to EE scores.

## 4. Discussion

The quality of nurses’ work environments during COVID-19 was operationalized using a series of questions on a variety of scales representing five domains shown in Figure 1. Regardless of the scale, nurses’ evaluations of different aspects of their workplace were classified into positive or negative perceptions or ratings. We found that nurses with negative ratings of most workplace safety indicators were more likely to suffer from PTSD, anxiety, and depression but not EE. Negative ratings of organizational support and workplace relations were associated with all of the adverse mental health outcomes included in this study. Nurses with negative ratings of organizational preparedness were more likely to report EE; and those experiencing staffing inadequacies were more likely to report all four of the adverse mental health outcomes. Finally, nurses working in environments with insufficient access to PPE were more likely to report PTSD and depression.

For this study, negative ratings on workplace safety indicators, such as fear of spreading COVID-19 from work to loved ones at home, were evidence of unsafe work environments. We found significant associations between nurses’ perceptions of unsafe workplaces and their self-reports of PTSD, anxiety, and depression. These findings are similar to pre-COVID-19 evidence where unsafe workplaces with frequent workplace violence negatively impacted nurses’ mental health and wellbeing [12,29,30,31]. A scoping review of 10 COVID-19 studies similarly found unsafe workplaces, characterized as inadequate infection control practices and policies, were associated with the development of mental health problems among nurses [4]. Other researchers specifically identified working in high-risk COVID-19 wards, fear of COVID-19 exposure, more frequent workplace exposure to the virus, and negative ratings of working safety while caring for COVID-19 patients as predictors of nurse anxiety and depression [32,33].

Surprisingly, workplace safety was unrelated to EE for the most part. This finding may be attributed to EE being the most important consequence of heavy workloads: EE may not be significantly influenced by workplace safety factors [34,35]. Pre-COVID 19 studies have linked heavy workload indicators to EE [36,37,38]. In this study, we also found that indicators of heavy workload—namely, staffing inadequacies, poor workplace relations (e.g., teamwork), and organizational support—were associated with higher levels of nurse EE.

In addition to workplace safety, access to sufficient resources and supplies in the workplace was negatively related to adverse mental health outcomes for the most part. This finding is consistent with pre-COVID 19 evidence linking inadequate nurse staffing to poor nurse and patient outcomes [13,18,36,37,38,39]. Most nursing research during COVID-19 has focused on the relationship between PPE adequacy and nurses’ mental health. These studies found that inadequate access to PPEs had negative implications for the mental health of healthcare workers, including nurses [40,41,42]. It is possible that staffing and PPE adequacy are equally important predictors of nurses’ mental health because of their influence on workplace safety. Although we found a non-significant relationship between adequate PPE and anxiety, it is possible this suggests that PPE adequacy does not contribute to nurse anxiety over and above the effect of other workplace predictors, such as staffing and workplace safety indicators. This is not to say insufficient access to PPE is not an anxiety provoking experience, but rather other constraints in nurses’ work environments such as limited workplace safety and inadequate staffing are more anxiety provoking for nurses.

In addition to resources and supplies, negative ratings of organizational support and workplace relations were associated with nurses’ experiences of PTSD, anxiety, and depression. Consistent with emerging COVID-19 evidence [32,33,43,44], this finding is attributed to the buffering effect of support—from the organization or the healthcare team—on work-related stress [45,46,47]. Nurses often rely on their team members and the support they receive from their organization to cope with workplace stressors [47]. Future research should more closely explore the mechanism by which various sources of support impact nurses’ mental health and wellbeing during a crisis.

Among organizational preparedness indicators, only frequent changes in COVID-19 policies and protocols were related to high PTSD, anxiety, and depression scores. It is possible that frequent changes in policies and protocols required nurses to quickly respond to and constantly adapt to the shifting landscape of COVID-19, a stress-provoking experience, especially in the context of a highly contagious infectious disease. Pre-COVID-19 evidence has linked feelings of stress with rapid and continuous changes in the workplace. This source of stress, known as “change fatigue”, is associated with adverse nurse outcomes, including EE [48,49,50,51].

Of note is that some COVID-19 workplace indicators were not important to nurse mental health in this study. Key examples include access to COVID-19 training, high-quality PPE, and N95 mask fitting. These non-significant findings could be attributed to the uncertain and limited understanding of COVID-19 pathophysiology and mode of transmission [52]. For example, after many months of controversy, the Public Health Agency of Canada only recently updated its COVID-19 guidelines on risk of airborne transmission of the virus requiring high-quality PPE, such as fitted N95 masks [53]. Future research should further explore with nurses why these indicators did not matter to them. These findings are worrisome given emerging pandemic evidence that suggests targeted COVID-19 training improves nurses’ mental health [33,54].

Even though the COVID-19 pandemic cannot be eliminated until a reliable vaccine is in place, nurses’ workplace conditions associated with adverse mental health outcomes can be purposefully and systematically addressed. Suboptimal work conditions pre-COVID-19 have been documented in the literature [13,19,55]. Nurse staffing inadequacies have been a particular concern with respect to impact on nurses, patients, and organizational outcomes [13,18,36,37,38,39,56]. Our study highlights how persistent staffing inadequacies may compound risk of adverse mental health outcomes for nurses. During the pandemic, as workplace stressors increase, we urge researchers, policy makers, and health employers to evaluate and optimize the conditions of nurses’ work environments as a strategy to prevent and mitigate adverse nurse mental health outcomes.

### Limitations

This is the first province-wide study in Canada to explore the impact of nurses’ workplace conditions during COVID-19 on the mental health of nearly 3700 nurses. Despite this strength, the low response rate raises concerns around sampling bias and generalizability. A comparison of our sample with the provincial nursing workforce based on Canadian Institute of Health Information showed our sample was closely representative of the provincial nursing population with respect to age, gender, professional designation, and employment status [57]. However, the generalizability of the findings to other samples and contexts may be limited. We also caution readers from establishing any cause-and-effect conclusions due to the cross-sectional nature of the study. Specifically, the stressor (the COVID-19 pandemic) occurred before an initial assessment of workplace conditions, meaning it cannot be concluded with certainty that the pandemic was the cause of the poor workplace conditions. It must also be considered that increased levels of nurse PTSD, anxiety, depression, and EE might worsen ratings of pre-existing issues with workplace conditions.

## 5. Conclusions

The findings of this study were consistent with a plethora of research evidence over the last two decades that repeatedly showed nurses’ workplace conditions are important to their experiences and their ability to deliver effective patient care. This study examined important aspects of nurses’ work environments unique to the context of COVID-19 and their impact on nurse PTSD, anxiety, depression, and EE. Some workplace conditions were more important than others. Workplace safety, access to resources and supplies, organizational support, and workplace relations most significantly influenced nurse mental health. The bottom line is nurses’ mental health can be improved through modifying the conditions of their work environments. Better practices and policies specifically addressing the above-listed workplace conditions are urgently needed to protect the health and safety of the nursing workforce, particularly during the time of COVID-19 crisis.

## Figures and Tables

**Table 1:** Descriptive statistics on nurse characteristics and workplace characteristics.

	Mean (SD)	*n*	%
Nurse Characteristics			
Role			
Direct care provider	-	3161	86
Nurse leader	-	393	10.7
Educator	-	122	3.3
Total		3676	
Designation ^1^			
RN	-	2735	74.4
RPN	-	200	5.4
Dually registered (RN/RPN)	-	15	0.4
LPN	-	714	19.4
Total		3664	
Years of nursing experience	12 (7.2)	-	-
Workplace Characteristics	Mean (SD)	*n*	%
Sector			
Acute care	-	2319	63.2
Community care	-	870	23.7
Long-term care	-	483	13.2
Total		3672	
Geographic area			
Urban	-	2324	63.6
Suburban	-	727	19.9
Rural	-	605	16.5
Total		3656	

^1^ Nurse designations: college-educated licensed practical nurse (LPN), baccalaureate-educated registered psychiatric nurse (RPN), and baccalaureate-educated registered nurse (RN).

**Table 2:** Descriptive statistics for key workplace condition predictors.

Workplace Conditions	*n*	Mean	SD	Range ^1^
Key predictors				
**Workplace safety**				
Frequency of direct contact with COVID patients ^1^	3587	2.91	1.97	1–7
Concern for contracting COVID ^2^	3597	3.47	1.12	1–5
Concern for bringing COVID home ^2^	3597	3.90	1.14	1–5
Likelihood of refusing unsafe work if denied appropriate PPE ^3^	3563	1.99	0.92	1–4
Confidence in ability to assess PPE requirements ^4^	3576	2.55	1.20	1–6
Confidence in ability to assess risk ^4^	3574	2.48	1.07	1–6
Experienced COVID-19 symptoms ^5^	3577	0.31	0.46	0–1
Submitted workers’ compensation ^5^	3549	0.02	0.12	0–1
**Resources and supplies**				
Nurse staffing adequacy ^6^	3597	3.48	1.60	1–6
PPE adequacy ^7^	3574	3.30	1.54	1–6
PPE quality ^7^	3569	3.59	1.50	1–6
Training ^8^	3526	2.33	0.99	1–5
N95 mask fitting ^9^	3575	3.27	1.54	1–7
**Organizational preparedness**				
Confidence in organizational handling of the pandemic ^4^	3527	3.49	1.34	1–6
Confidence in manager’s handling of the pandemic ^4^	3522	3.48	1.46	1–6
Frequency of COVID-19 related policy changes ^10^	3527	5.47	1.68	1–8
Transparency of pandemic-related organizational decisions ^11^	3522	3.29	1.02	1–5
**Organizational support**				
Told to work despite exposure ^5^	3571	0.24	0.43	0–1
Told to work despite symptoms ^5^	3556	0.31	0.23	0–1
Organizational support ^12^	3523	2.64	1.03	1–5
**Workplace relations**				
Relationships with nursing colleagues ^13^	3524	3.05	0.79	1–5
Relationships with manager ^13^	3526	3.25	0.80	1–5
Relationships with the rest of allied health ^13^	3523	3.11	0.70	1–5

Note: ^1^ (3 = “once a month”); ^2^ (3 = “somewhat concerned”, 4 = “very concerned”); ^3^ (2 = “likely”); ^4^ (2 = “confident” 3 = “somewhat confident”, 4 = “slightly confident”); ^5^ (0 = “no”, 1 = “yes”); ^6^ (3 = “slightly adequate”, 4= “slightly inadequate”); ^7^ (3 = “somewhat agree”, 4 = “somewhat disagree”); ^8^ (2 = “somewhat adequate”); ^9^ (3 = “within the last year”); ^10^ (5 = “once a week”); ^11^ (3 = “fair”); ^12^ (3 = “slightly supported”); ^13^ (3 = “stayed the same”). The complete response options and coding schemes for all items can be found in Figure 1.

**Table 3:** Descriptive statistics for mental health outcomes and proportions for cutoff categories.

Outcome Variables	Frequency	Percent	Mean	SD	Range
Post-traumatic stress disorder **(PTSD) (PTSS-14)**					
Under cutoff (14–44)	1944	52.9			
Met cutoff, PTSD (45–98)	1732	47.1			
Total	3369		46.68	19.37	14–98
**Anxiety (GAD-7)**					
Under cutoff (0–9)	2114	62.4			
Met cutoff, anxiety (10–21)	1273	37.6			
Total	3387		8.53	5.80	0–21
**Depression (PHQ-9)**					
Under cutoff (0–9)	1972	58.6			
Met cutoff, major depression (10–27)	1391	41.4			
Total	3363		8.99	6.23	0–27
**Emotional Exhaustion (EE) (MBI-HSS)**					
Low EE (0–16)	602	18.4			
Moderate EE (17–26)	692	21.2			
High EE (27–54)	1975	60.4			
Total	3269		30.05	13.23	0–54

**Table 4:** Final linear regression models regressing PTSD, anxiety, depression, and EE measure sum scores on demographic control and workplace condition variables.

	PTSD (PTSS-14)			Anxiety (GAD-7)			Depression (PHQ-9)			Emotional Exhaustion (MBI-HSS)		
Variables	*B*	95% CI	*p*	*B*	95% CI	*p*	*B*	95% CI	*p*	*B*	95% CI	*p*
(Constant)	−9.50	−14.35–−4.65	0.00	−6.46	−7.94–−4.98	0.00	−5.43	−7.06–−3.79	0.00	−1.92	−5.27–1.43	0.26
**CONTROL VARIABLES**												
Role (Direct care provider)	−1.23	−2.96–0.49	0.16	−0.61	−1.14–−0.09	0.02	−0.55	−1.13–0.03	0.06	−3.29	−4.48–−2.10	0.00
Sector (Acute care)	1.04	−0.26–2.34	0.12	−0.04	−0.44–0.36	0.84	−0.19	−0.63–0.25	0.39	0.42	−0.48–1.32	0.36
Geographic area (Urban/suburban)	0.94	−0.62–2.49	0.24	−0.07	−0.54–0.41	0.79	0.00	−0.53–0.52	0.99	−0.20	−1.27–0.87	0.71
Designation (RN/RPN)	0.55	−0.96–2.07	0.47	0.52	0.06–0.98	0.03	0.04	−0.47–0.55	0.87	1.47	0.42–2.52	0.01
Experience (Years of experience)	−0.08	−0.17–0.00	0.06	−0.06	−0.09–−0.04	0.00	−0.04	−0.06–−0.01	0.01	−0.09	−0.15–−0.03	0.00
**KEY PREDICTORS**												
**Workplace safety**												
Frequency of direct contact	0.62	0.30–0.94	0.00	0.09	0.00–0.19	0.06	0.10	−0.01–0.21	0.07	0.38	0.16–0.60	0.00
Concern for contracting COVID	1.27	0.48–2.06	0.00	0.37	0.13–0.61	0.00	0.16	−0.11–0.42	0.24	0.47	−0.07–1.01	0.09
Concern for bringing COVID home	1.17	0.40–1.95	0.00	0.44	0.21–0.68	0.00	0.30	0.04–0.56	0.02	0.29	−0.24–0.82	0.29
Refusing unsafe work if denied appropriate PPE	1.56	0.91–2.21	0.00	0.44	0.25–0.64	0.00	0.50	0.29–0.72	0.00	0.37	−0.08–0.82	0.11
Experienced COVID-19 symptoms	4.12	2.87–5.37	0.00	1.24	0.86–1.63	0.00	1.53	1.11–1.95	0.00	1.81	0.94–2.67	0.00
**Resources and supplies**												
Nurse staffing adequacy	1.03	0.62–1.44	0.00	0.32	0.20–0.44	0.00	0.32	0.19–0.46	0.00	1.41	1.13–1.69	0.00
PPE access adequacy	0.77	0.31–1.23	0.00	0.08	−0.06–0.22	0.27	0.16	0.00–0.31	0.05	0.25	−0.07–0.57	0.12
**Organizational preparedness**												
Confidence in organizational handling of the pandemic	0.63	0.03–1.29	0.06	0.17	−0.03–0.37	0.09	0.11	−0.12–0.33	0.35	0.77	0.32–1.23	0.00
Frequency of COVID-19 related policy changes	1.30	0.94–1.66	0.00	0.43	0.32–0.54	0.00	0.46	0.34–0.59	0.00	0.90	0.65–1.15	0.00
Transparency of pandemic-related organizational decisions	0.67	−0.14–1.49	0.10	0.11	−0.13–0.36	0.37	0.18	−0.09–0.45	0.20	0.68	0.12–1.24	0.02
**Organizational support**												
Organizational support	1.97	1.13–2.80	0.00	0.60	0.35–0.86	0.00	0.71	0.43–0.99	0.00	1.48	0.90–2.06	0.00
**Workplace relations**												
Relationships with nursing colleagues	2.12	1.28–2.96	0.00	0.73	0.47–0.99	0.00	0.74	0.46–1.03	0.00	1.03	0.45–1.61	0.00
Relationships with manager	1.34	0.44–2.23	0.00	0.35	0.08–0.62	0.01	0.15	−0.15–0.45	0.33	0.87	0.25–1.49	0.01
Relationships with the rest of allied health	2.44	1.49–3.39	0.00	0.56	0.27–0.85	0.00	0.71	0.39–1.03	0.00	1.31	0.66–1.97	0.00
Final model *F*-statistic, explained variance (Adjusted *R*^2^), model *p*	59.98 (19, 3229)	25.7%	0.00	51.40 (19, 3245)	22.7%	0.00	40.92 (19, 3223)	19.0%	0.00	58.51 (19, 3136)	25.7%	0.00

## Data Availability

The data presented in this study are available on request from the corresponding author. The data are not publicly available due to ethical and privacy restrictions.
